# SSVEP phase synchronies and propagation during repetitive visual stimulation at high frequencies

**DOI:** 10.1038/s41598-021-83795-9

**Published:** 2021-03-02

**Authors:** Tsvetomira Tsoneva, Gary Garcia-Molina, Peter Desain

**Affiliations:** 1 Brain, Behaviour and Cognition Department, Philips Research, High Tech Campus 34, 5656 AE Eindhoven, The Netherlands; 2 Donders Institute for Brain, Cognition and Behaviour: Centre for Cognition, Radboud University, Montessorilaan 3, 6525 HR Nijmegen, The Netherlands; 3 Sleep Number Labs, 111 N Market St, San Jose, CA 95113 USA; 4 Center for Sleep and Consciousness, University of Wisconsin, Madison, WI 53719 USA

**Keywords:** Visual system, Sensory processing

## Abstract

Steady-state visual evoked potentials (SSVEPs), the brain response to visual flicker stimulation, have proven beneficial in both research and clinical applications. Despite the practical advantages of stimulation at high frequencies in terms of visual comfort and safety, high frequency-SSVEPs have not received enough attention and little is known about the mechanisms behind their generation and propagation in time and space. In this study, we investigated the origin and propagation of SSVEPs in the gamma frequency band (40–60 Hz) by studying the dynamic properties of EEG in 32 subjects. Using low-resolution brain electromagnetic tomography (sLORETA) we identified the cortical sources involved in SSVEP generation in that frequency range to be in the primary visual cortex, Brodmann areas 17, 18 and 19 with minor contribution from sources in central and frontal sites. We investigated the SSVEP propagation as measured on the scalp in the framework of the existing theories regarding the neurophysiological mechanism through which the SSVEP spreads through the cortex. We found a progressive phase shift from posterior parieto-occipital sites over the cortex with a phase velocity of approx. 8–14 m/s and wavelength of about 21 and 24 cm. The SSVEP spatial properties appear sensitive to input frequency with higher stimulation frequencies showing a faster propagation speed.

## Introduction

Repetitive visual stimulation (RVS), or visual flicker, at a frequency in the range from 1 to 100 Hz elicits an oscillatory response in the electroencephalogram (EEG) at the same frequency and/or its harmonics known as steady-state visual evoked potentials (SSVEPs). This response is time and phase-locked to the driving stimulus. It appears most prominently over parietal and occipital areas as they are closer to the visual information processing centers of the brain cortex but can also be detected in central and frontal areas^1^.

Because of their high signal to noise ratio and robustness to artifacts, SSVEPs have proven beneficial in both research and clinical applications.

The wide spatial extent of the SSVEPs across the scalp can provide means for studying the collective behaviour of large groups of neurons^2^ allowing the investigation of neural processes underlying brain rhythmic activity and functional brain connectivity. When RVS is superimposed on a cognitive task, especially around the alpha frequency band (8–12 Hz), specific topographic changes in amplitude and phase of the SSVEP appear analogous to the site-specific reductions in alpha EEG amplitude associated with cognitive and motor tasks^3^. This technique is known as steady-state topography and has been applied for studying working memory^3,4^, binocular rivalry^5^, selective attention^6,7^, vigilance^8^ and even emotions^9^.

SSVEPs are also used as a diagnostic tool to study pathological brain dynamics and as an investigative probe of the cortical mechanisms of visual perception. The amplitude and phase of the SSVEP vary as a function of stimulus parameters such as temporal and spatial frequency, contrast, luminance, hue, visual angle and spatial attention^1,10–13^ and can be a sensitive electrophysiological index of a variety of visual-perceptual and cognitive functions^14^. SSVEP-based techniques such as photic driving and sweep-VEP are used for evaluating visual acuity of infants and children^15^, studying infant vision^16^, assessment of neuro-visual function^17^, spatial neglect^18^ and epilepsy^19^. SSVEPs are also applied for the investigation and treatment monitoring of age-related and neurodegenerative disorders, such as Alzheimer’s disease, Parkinson, Schizophrenia and others^8,20,21^. They could also be an effective diagnostic tool for detecting or predicting age-related cognitive decline^22^.

Lately the topic of controlled entrainment of perceptually relevant brain oscillations by non-invasive rhythmic stimulation in a frequency-specific manner has become increasingly popular in research of brain rhythms^23^. Peripheral stimulation, including sensory and transcranial stimulation, could be used to probe the causal role of oscillatory brain activity on cognition and emotion. To show causality here, we need to verify that evoked oscillatory signatures, which are already well-studied correlates of particular behaviour outcome, do give rise to a similar behaviour outcome^23^. A number of groups has shown a promising behavioral and electrophysiological evidence that external rhythmic stimulation protocols may change attention and perception^24,25^, affect memory^26^, enhance sleep^27^ and even attenuate Alzheimer’s disease-associated pathologies^28^ by potentially reducing amyloid load^29^.

SSVEPs are also widely used in overt and covert attention based brain–computer interfaces (BCIs). In BCIs visual stimuli having distinctive properties (e.g., frequency or phase) are simultaneously presented to a user who selects among several commands by focusing his/her attention on the corresponding stimulus. Most current SSVEP-based BCIs use frequencies between 5 and 40 Hz, which may cause discomfort and induce fatigue. A safety risk also exists because RVS in that range may cause epileptic seizures^30^ or headache and eye strain complaints^31^ and, therefore, higher frequencies are preferable. The signal-to-noise ratio of HF-SSVEP (high frequency-SSVEP) is as high as that of LF-SSVEP (low frequency-SSVEP), which makes HF-SSVEP an ideal candidate for practical BCIs^32,33^ due to the minimal or even absence of user discomfort vis-a-vis the flicker^34^. HF-SSVEP elicited by stimulation frequencies beyond the critical perception frequency have been successfully used for BCI applications^35^. SSVEPs elicited by frequencies that are only 0.2 Hz apart from each other can be distinguished in the EEG^36^.

The broad range of SSVEP applications in research and clinical practice come with their requirements regarding safety, comfort and ease of use. Higher stimulation frequencies and sparse electrode arrays are often preferred as a more practical alternative. This could potentially speed up system preparation, increase the amount of people tested, and avoid known side effects of prolonged visual stimulation. Here, care should be taken translating existing knowledge regarding the SSVEP phenomena and better understanding of the spread and the propagation of the response in scalp recorded EEG is needed.

Despite the years of investigation, the mechanisms behind SSVEP generation are still not fully understood. Vialatte et al.^37^ identify three concurrent theories behind SSVEP origin and propagation. According to the first one SSVEPs originate in the primary visual cortex and propagate by the combined activity of locally and broadly distributed sources^38^. The second one argues that SSVEPs are generated by a finite number of electrical dipoles that are activated sequentially in time, starting with a dipole located in the primary visual areas^14^. While isolated dipoles account for the strongest activity, they may not sufficiently describe the complex distribution of electrical activity in the brain, thus, the third theory^2^ suggests that VEPs originate in the primary visual cortex, and propagate to other brain areas through cortical travelling and standing waves. These theories, do have in common the fact that SSVEPs propagate, starting from the primary visual cortex, and involve more than one single source dipole.

Cortical activation of steady-state and transient visual stimuli appears to be similar, with a consensus that early visual components originate in the primary visual cortex (Brodmann’s area 17, striate cortex, V1)^14^. A visual response then generally follows the hierarchical organization of cortical areas from V1 to V2, V3^39^. Co-localization of dipole locations with fMRI activation sites shows that the two major sources of the SSVEP are located in primary visual cortex (V1) and in the motion sensitive (MT/V5) areas with minor contributions from mid-occipital (V3A) and ventral occipital (V4/V8) areas^14^.

Cortical propagation of oscillatory brain activity has been investigated in various animal and human studies both with intracranial and transcranial recordings^40^. This wave-like dynamic behaviour has been observed for brain oscillation in the alpha (8–12 Hz), theta (4–8 Hz)^40–42^, and sleep delta (0.5–4 Hz)^43,44^ bands as well as visually evoked potentials^2,38,45–47^. Early reports of travelling waves state that the estimated speed of travelling over the scalp generally varies between 1 and 20 m/s^48^. Alpha rhythm phase velocities from 3 to 7 m/s^49^ have been estimated over the scalp (6–14 m/s when corrected for cortical folding^50^), with corrected for folding lower alpha ($\sim$ 8 Hz) velocity around 6.4 m/s and upper alpha ($\sim$ 12 Hz) velocity around 8.4 m/s. Alpha waves have been also observed in ECoG recordings at phase velocity between 0.7 and 2 m/s^40^. The propagation velocity of theta waves moving through the hippocampus in ECoG recordings seem to be between 1 and 5 m/s^42^. In sleep, slow waves propagation at a speed from 1 to 7 m/s^43^ and from 0.4 to 6.3 m/s^44^ have also been observed. When it comes to cortical propagation of evoked oscillations, analyzing topographical phase relationships of the ERP P1 component (evoked by the Stroop task), having a frequency characteristics primarily in the alpha range, reveals a systematic posterior to anterior traveling pattern at a speed of approx. 3 m/s (or approx. 6 m/s if corrected for cortical folding)^47^. EEG activity following eye movement also show a wave propagation dynamics with mean velocity from 0.2 m/s at 0.5 Hz to 14 m/s at 32 Hz^51^. For SSVEPs, scalp phase velocities for checkerboard stimulation around the alpha peak have been estimated between 7 and 11 m/s (corrected for cortical folding using a folding factor of 2.2)^2^. SSVEPs elicited by a circular black/white checkerboard pattern at frequencies between 2 and 20 Hz, have showed a traveling wave behaviour propagating from occipital to prefrontal electrodes in the upper alpha band (phase velocities ranging from about 2.4 to 3.3 m/s along the scalp), but not in beta band^46^. Another study^38^ using stimulation between 3 and 30 Hz, also presents evidence for traveling waves (at a speed of   1–3 m/s) at frequencies in the upper alpha band (11–13 Hz) but not in delta and lower alpha.

While it might be difficult to dissociate between the different theories behind SSVEP origin and propagation, understanding the cortical properties of this response as measured by the EEG is crucial for the design and implementation of the practical applications identified above. Sparse electrode montages and higher frequencies positively impact the ease of use, patient throughput, comfort and safety. In this study we aim at investigating the spatiotemporal oscillatory properties of SSVEPs. Particularly, SSVEP responses at high frequencies, e.g. in the gamma band (40–60 Hz), which have not received enough attention and little is known about their generation and factors affecting the response. We set to investigate their site of origin and their cortical propagation properties in terms of speed and phase.

## Results

### SSVEP response

**Figure 1:**
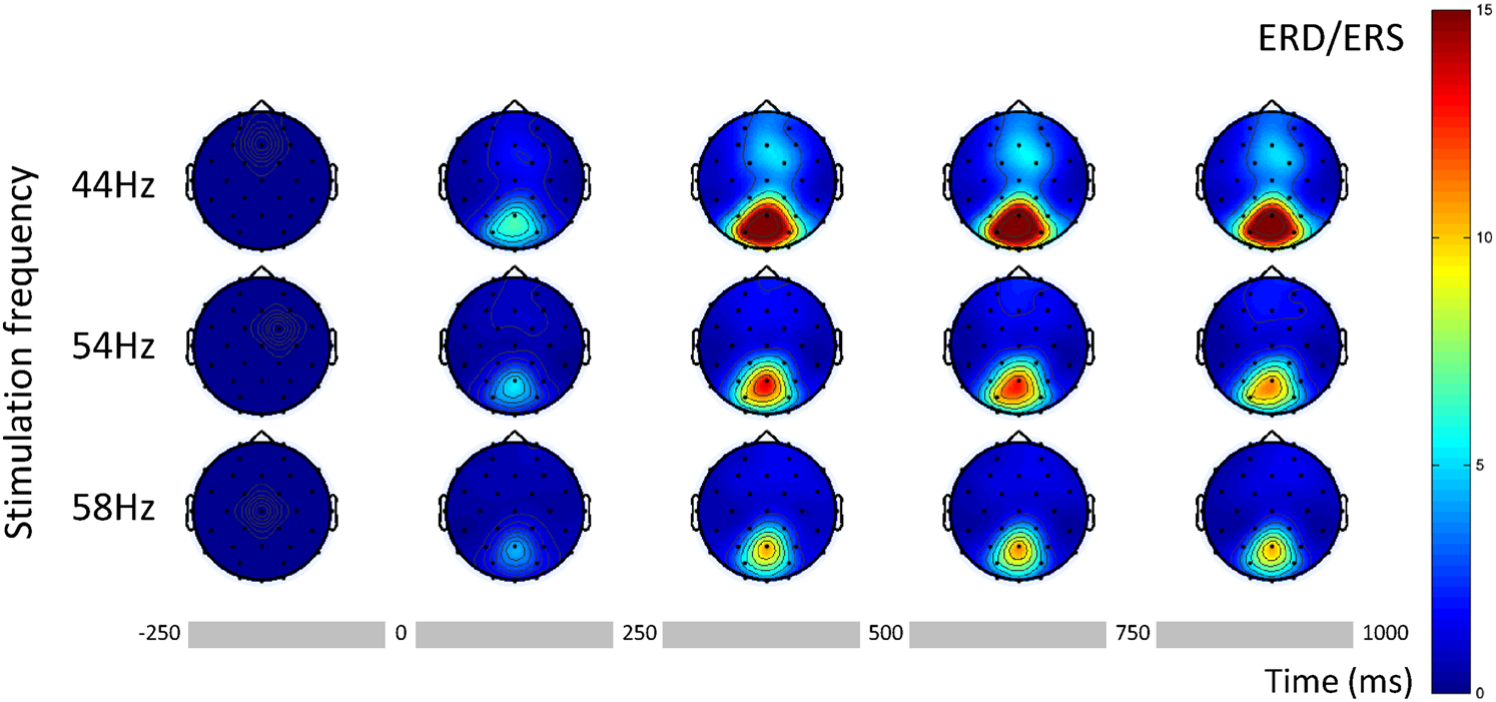
ERS at the fundamental frequency for RVS at 44, 54 and 58 Hz in 250 ms long windows starting 250 ms before stimulation onset until 1000 ms after. Stimulation onset is at time 0, we take the 1st window [-250 0) ms as reference.

Time frequency decomposition analysis revealed a stable SSVEP response starting within 250 ms from stimulus onset at each fundamental and its harmonic frequencies. Figure 1 shows the percentage power change (event related synchronization, ERS) for the first second of stimulation for three of the conditions at each fundamental frequency. As expected the response is stronger over parieto-occipital sites. Lower frequency conditions are characterized by a broader response as compared to higher frequencies, showing some contribution from fronto-central sites. In all cases, after the transient response the first 250 ms, we observe a sustained steady state response for the whole duration of stimulation. These observations were consistent across all stimulation conditions.

For a more comprehensive overview of the neural dynamics at the fundamental and the harmonic frequencies of this dataset as well as the interaction with ongoing oscillatory activity see Tsoneva et al.^52^.

### Source analysis

**Figure 2:**
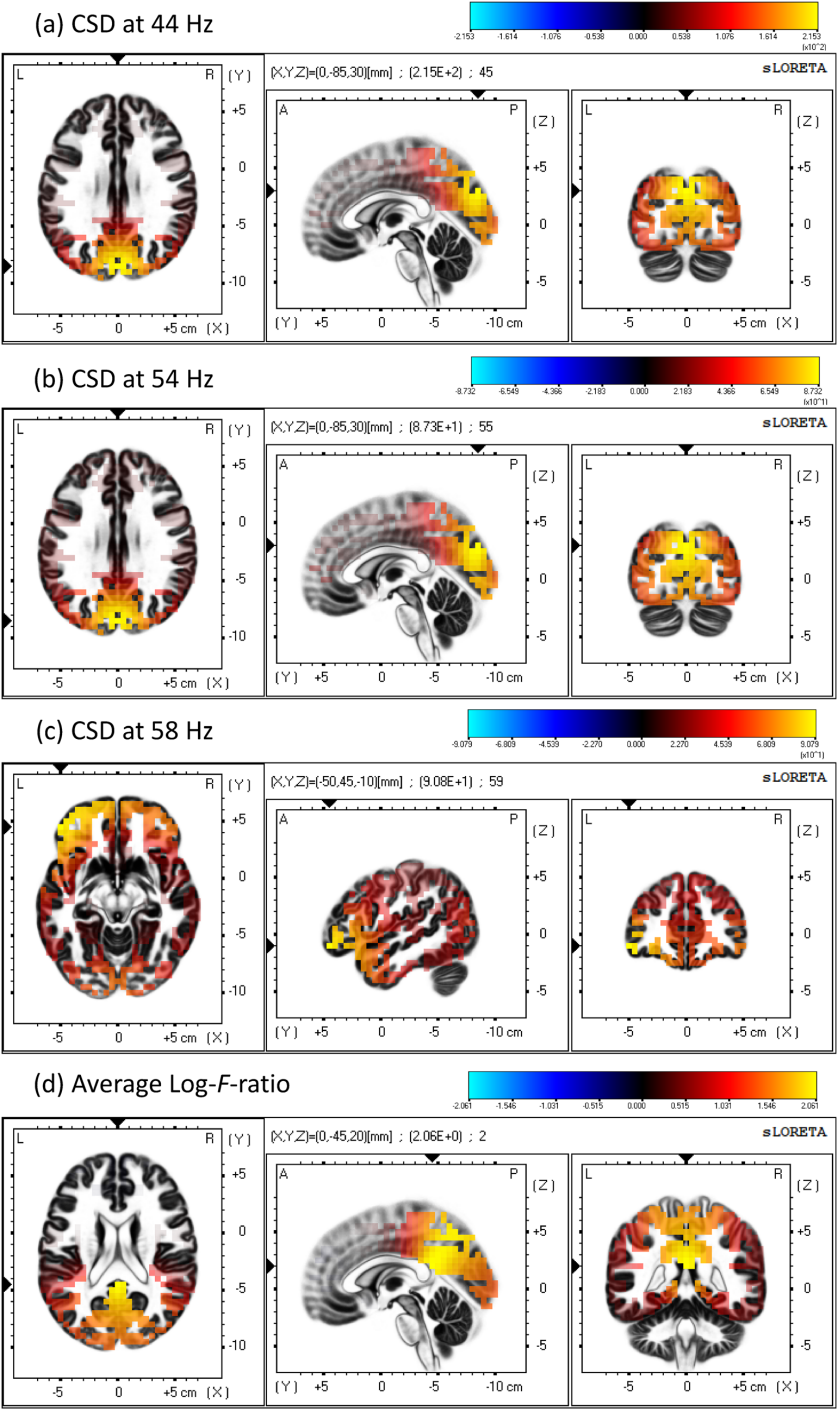
Low-resolution brain electromagnetic tomography (sLORETA) at the fundamental frequency component under diverse stimulation conditions: (**a**) 44 Hz, (**b**) 54 Hz, (**c**) 58 Hz. (**d**) The average log F-ratio statistics across all fundamental frequency components. The squared magnitude of the CSD and the average log F-ratio are colour coded from blue (minimum) through grey (zero) to red and bright yellow (maximum). Slices from left to right: axial (viewed from top), sagittal (viewed from left), and coronal (viewed from back). *L* left, *R* right, *A* anterior, *P* posterior.

To identify the spread of cortical sources involved in SSVEP generation we performed source localization analysis using the sLORETA software. This analysis revealed strongest activation in response to frequencies between 40 and 56 Hz at coordinates: $X = 0 , Y = - 85 , Z = 30$, identified as the Cuneus in Brodmann area 19 of the Occipital cortex (see Fig. 2a,b). Other areas of the visual cortex such as V1 (Brodmann area 17) and V2 (Brodmann area 18) were also identified as contributors. Additionally, minor contribution from sources in central and frontal sites could be observed. These coordinates were consistent across all conditions under 56 Hz for both, the fundamental and subharmonic components (see Supplementary information Figure S1ab). We did not find a consistent activation for higher order harmonic frequencies.

The source distribution for stimulation at 58 Hz and 60 Hz was slightly different (see Fig. 2c). While activity in the visual cortex was present, the strongest activation was found in the inferior frontal gyrus (Brodmann area 47) for the fundamental frequencies and middle frontal gyrus (Brodmann area 11) for the sub-harmonic frequencies (see Supplementary information Figure S1c). Statistical testing, however, did not show a significantly higher current source density (CSD) for those coordinates when compared to the second before stimulation onset as baseline.

The average log-F-ratio statistics was quite consistent across all stimulation conditions highlighting cortical areas in the occipital, parietal, limbic and frontal lobe. The average log-F-ratio across all conditions is shown in Fig. 2d. The log-F-ratio thresholds for $p < 0.01$ and $p < 0.05$ for the fundamental component at each stimulation condition are listed in Table 1. The results for the sub-harmonic components of each stimulation condition can be found in the Supplementary information Figure S1d and Table S1. Higher order harmonics did not show any significant difference from baseline (the second before stimulation onset).

**Table 1:** Log-F-ratio thresholds for the fundamental component at each stimulation condition.

Stimulation condition	F($p<0.01$)	F($p<0.05$)
40	1.448	1.203
42	1.452	1.146
44	1.362	1.067
46	1.619	1.537
48	1.382	1.089
52	1.006	0.939
54	1.153	0.891
56	1.417	1.364
58	1.063	1.012
60	0.863	0.805

### Source of origin

**Figure 3:**
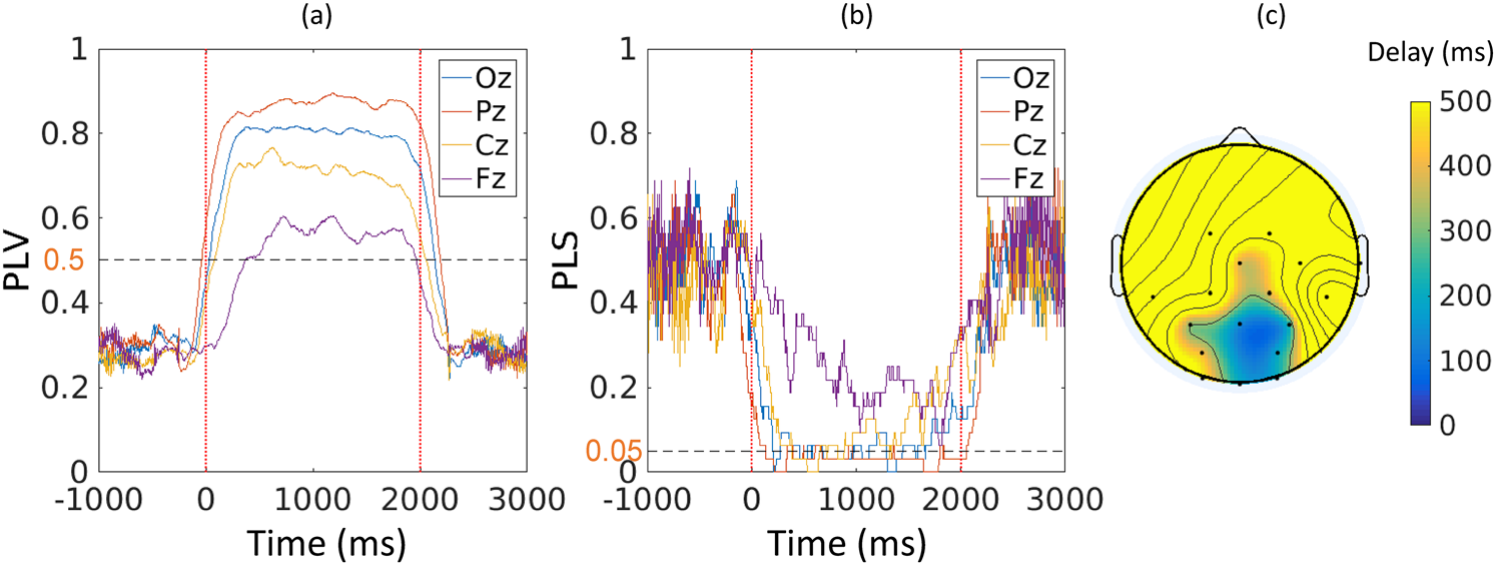
Phase locking between the light stimulation signal and the EEG signals for RVS at 44 Hz. (**a**) Instantaneous PLV between electrode locations along the midline and the light stimulation signal. The vertical red dotted lines mark the onset and offset of stimulation. The black dashed line marks the PLV threshold. (**b**) PLS for electrode locations along the midline and the stimulation signal. The vertical red dotted lines mark the onset and offset of stimulation. The black dashed line marks the PLS threshold. (**c**) Spatial distribution of phase-locking delays.

While LORETA is very useful for localizing the activity, it cannot tell us about the chronology of events. Generally, the SSVEP response is time- and phase-locked to the driving stimulus. Figure 3a shows the instantaneous phase locking value (PLV) between midline electrodes and the stimulation for one of the stimulation conditions (44 Hz) averaged over all subjects. Phase locking ($PLV > 0.5$) occurs already within the first 150 ms and saturates after approximately 250 ms, following the transient response. The same behaviour was observed for all other conditions. Because we are particularly interested in the phase properties of the response, we use finite impulse response (FIR) filters at the stimulation frequency, which have zero phase distortion after correcting for their linear delay. The inherently high order and the equiripple nature of those filters, however, can cause discontinuities at the head and tail of their impulse response. This is mostly visible at the start and the end of stimulation where the steady-state response is still weak. To deal with this issue we only consider PLVs for samples where the phase locking statistic (PLS) show significance of the phase covariance ($PLS < 0.05$). Figure 3b shows the decrease of PLS shortly after the stimulus onset and its relatively low value for the whole stimulation period, especially for parieto-occipital channels.

To identify the initial source locking to the driving stimulus first, we look at the significant $PLVs > 0.5$ for all channels. The locking delays for different channels at stimulation frequency of 44 Hz are visualized in Fig. 3c. Note that for some channels PLS never reaches significance level. As expected, the channels with minimum locking delays are in the parietal and occipital areas for all frequencies, with channel Pz being the first to lock for 7 out of the 10 frequencies. For consistency, henceforth, we select channel Pz as source of wave propagation.

### Phase velocity

**Figure 4:**
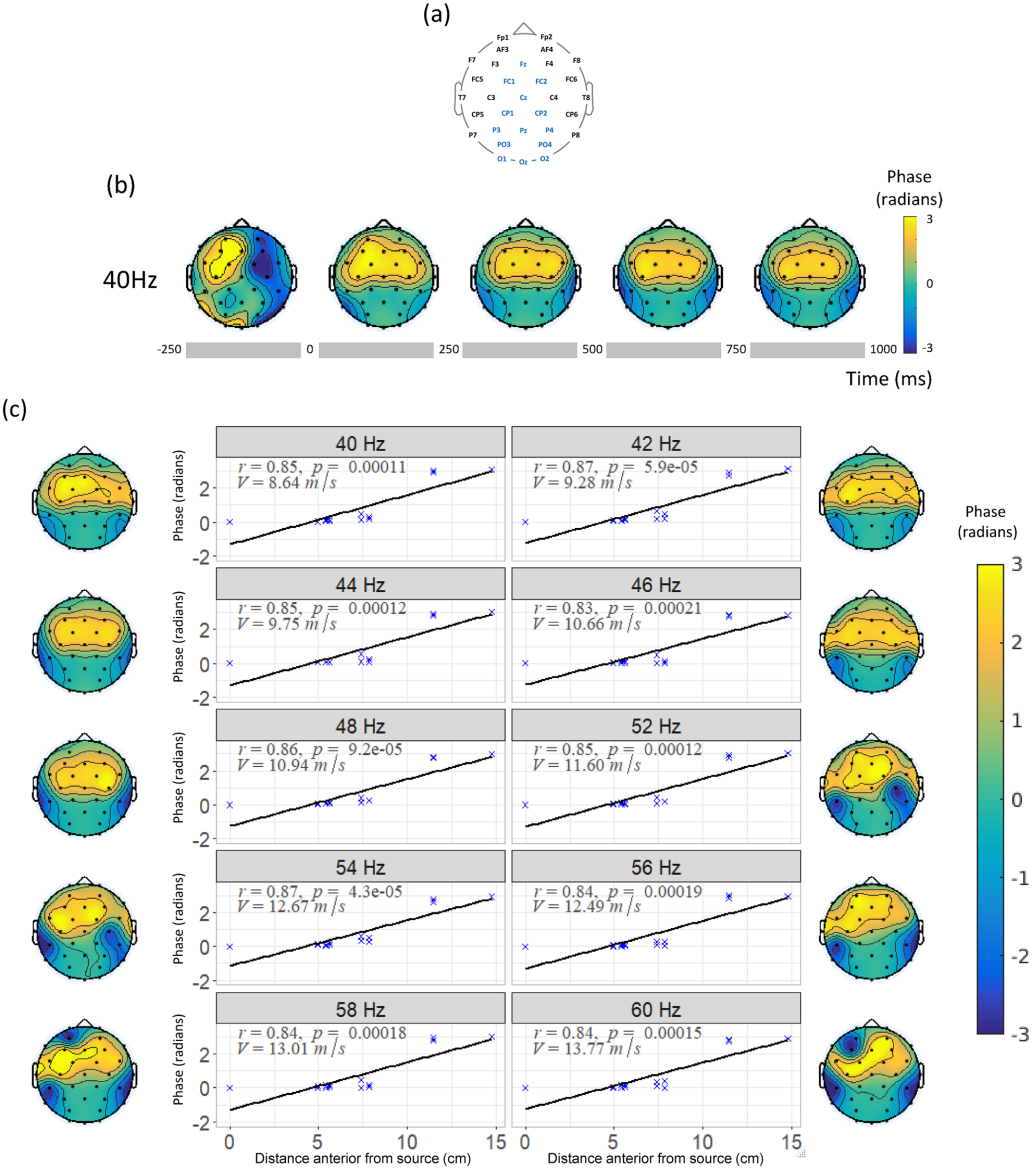
Phase velocity estimation. (**a**) Electrode positions and the selected set of electrodes for phase velocity estimation (in blue). (**b**) Average phase difference between the source (Pz) and all electrode locations for stimulation at 44 Hz over time starting 250 ms before stimulation until 1000 ms after stimulation onset. (**c**) Topography of phase differences with respect to the source location Pz, 250 ms after stimulus onset, and the phase gradient as estimated by a first order polynomial fit for the selected electrode set for each stimulation condition in different subplots. Correlation coefficients (*r*), p values (*p*) and phase velocity estimates (*V*) are reported for each stimulation condition.

The phase difference between each electrode and the source appears to be constant for the whole stimulation period (see Fig. 4b). The value, calculated over 5 cycles for each frequency and averaged over all trials and all subjects every 250 ms shows a progressive phase shift in the EEG, suggesting spherical propagation in all directions from the source.

To characterise the phase velocity of the propagation we estimate the spatial phase gradients by fitting a first order polynomial to the phase estimates for a set of electrodes covering the sites identified in the source analysis, namely O1, O2, Oz, PO3, PO4, P3, Pz, P4, CP1, CP2, Cz, FC1, FC2 and Fz as shown in Fig. 4a ordered in increasing distance from the source Pz.

The phase velocity is inversely proportional to the phase gradient and is calculated using Eq. (6) adjusting for the stimulation frequency for each condition. Figure 4c shows the first order polynomial fit and the phase velocity estimates over the area identified by the source analysis for all conditions 250 ms after stimulus onset when the steady-state response should be already stable. The correlation between phase difference and electrode distance (see Eq. 5) is significant for all conditions with mean correlation coefficient $r = 0.85 (\pm 0.013)$ and mean p = 0.001 (± 5.5265.526e−05) across conditions. The estimated velocity ranges between 8.64 and 13.77 m/s. These estimates suggest waves propagating at wavelengths between 21.6 and 23.5 cm across all conditions.

**Figure 5:**
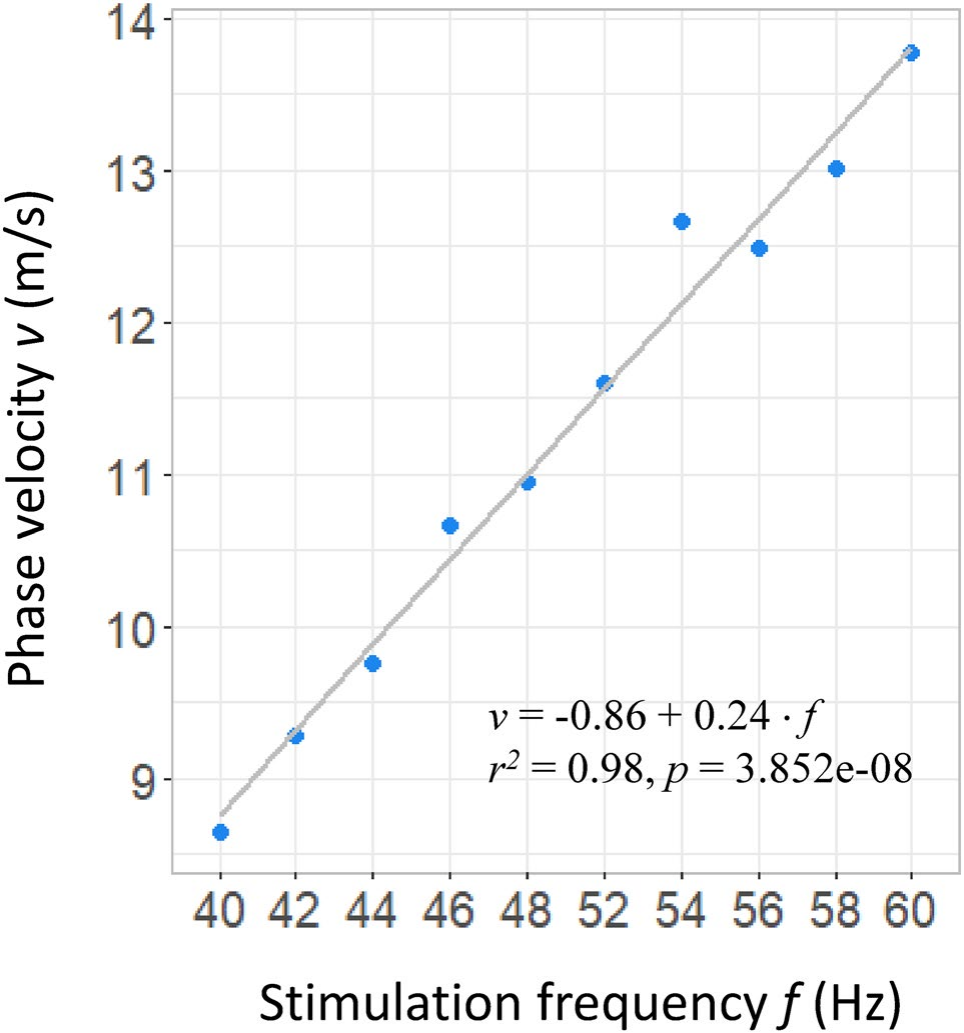
Phase velocity as a function of stimulation frequency. The black line shows the first order polynomial fit with correlation coefficient $r = 0.99$ and $p = 3.852e-08$.

The phase velocity estimates are by definition sensitive to stimulation frequency (see Eq. 6). Figure 5 shows a linear relationship and a significant positive correlation between our velocity estimates and frequency of stimulation ($r = 0.99$, $p = 3.852e-08$). We also estimate the linear regression model parameters that describe this relationship. An increase in the stimulation frequency of 1 Hz results in about 0.24 m/s increase in phase velocity. It is important to note that the validity of this model applies to the range of frequency tested as different mechanisms might apply to oscillations in different frequency sub-systems.

## Discussion

We set to investigate the SSVEP responses to periodic visual stimulation at high frequencies within the gamma band (40–60 Hz) by studying the dynamic properties of EEG measured by sparse electrode montage in 32 subjects. Not surprisingly the strongest cortical sources involved in SSVEP generation, identified by the sLORETA analysis, were found in the primary visual cortex, Brodmann areas 17, 18 and 19. Minor contribution from sources in central and frontal sites could also be observed. Looking at the phase response we found phase locking occurring already within the first 150 ms with channel Pz being the one that synchronized to the driving stimuli first for the majority of the tested conditions. While it is difficult to distinguish between the neurophysiological mechanism through which the SSVEP spreads through the cortex, being a finite number of electrical dipoles that are activated sequentially in time, or through cortical travelling waves, the SSVEP response as measured by the surface EEG shows a progressive phase shift in all directions from the source. Our results suggest that high-frequency-evoked SSVEPs propagate over the cortex with a phase velocity of approx. 8–14 m/s and wavelength of about 21 and 24 cm.

It should be emphasized that estimates of phase velocity depend on a variety of different factors. Some authors highlight the fact that the choice of EEG recording reference might play an important role with bipolar recordings giving lower estimates as compared to reference methods^2,49^. Other estimates are based on reference-free methods such as surface Laplacian methods^38,49^. MEG and ECoG recordings produce lower velocity estimates than EEG measurements^40,46^. Apart from the above mentioned factors affecting the phase estimates, inter-electrode distance could also vary. Some authors choose to correct for cortical folding (fissures)^2,53^, while others do not^40,43^. Furthermore, using euclidian or great-circle distance between electrodes results in different velocity estimates. All these factors inherently affect the phase velocity estimates making it difficult to compare between studies.

There is also lack of literature reporting SSVEP propagation in the gamma band. However, frequency seems to be an important factor and a number of authors show that SSVEP properties are sensitive to flicker frequency^38,46^. Sleep slow waves (0.5–4 Hz) are reported to be travelling at 1–7 m/s, with most of the scalp velocities being in the 2–3 m/s range^43^. Alpha waves seem to be travelling at 3–8 m/s, with lower alpha (8 Hz) around 6.4 m/s and upper alpha (12 Hz) around 8.4 m/s^49^. One of the few studies looking at higher frequencies reports phase velocities ranging from 0.2 m/s at 0.5 Hz to 14 m/s at 32*Hz* for eye-movement evoked responses. This progressive increase of velocity with the frequency of oscillations (spontaneous or evoked) seems to be in line with our estimates and the correlation with frequency of stimulation we found (see Fig. 5). It is, however, important to note that SSVEPs are sensitive to cortical resonances in at least three frequency subsystems^10,38^ and the mechanisms that apply at higher frequencies might not directly translate to oscillations at lower frequencies.

In their theoretical framework, considering the rough boundaries of propagation velocities within cortico-cortical fibers and accounting for experimental and theoretical uncertainties, some authors^53^ expect the phase velocity of cortical waves traveling away from primary visual areas to not exceed 30 m/s (up to a factor of three from observed estimates between 6 and 9 m/s). Our estimates of SSVEP propagation speed using Laplacian derivation and great-circle distance are within these loose boundaries.

**Figure 6:**
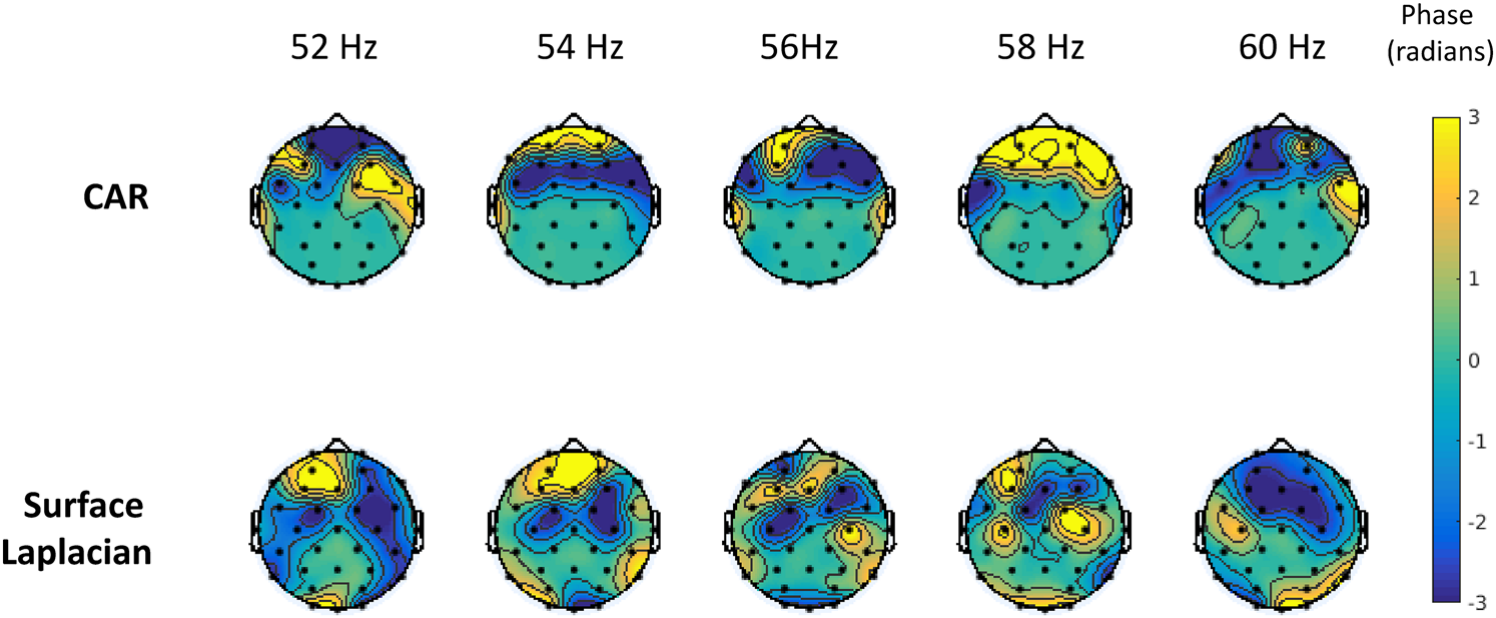
Topography of phase differences with respect to the source location Pz for a subset of the stimulation condition in the lack of stimulation (350 ms before stimulation onset) in the same interval using CAR and surface Laplacian.

Scalp recorded patterns of cortical activity also reflect volume conduction across the scalp, skull and other tissues. Volume conduction is especially a problem of reference EEG recordings, which are known to overestimate propagation velocities. Alternatively, close bipolar electrodes generally do better, but may be biased toward shorter wavelengths^54^. In this study, the spatial distortion from conductivity variations was minimized by applying a surface Laplacian, which is reference independent linear data transformation that maintains the invariant aspects of the EEG signal and provides means for obtaining continuous estimates of radial current flow at the scalp for both low- and high-density EEG montages^55^. It enhances the spatial information of the EEG signal by emphasizing sources within 2–3 cm of the electrode and providing good representation of the underlying current dipoles^38^. In our initial analysis we compared two preprocessing approaches, namely the widely used common average reference (CAR) and surface Laplacian. Signals preprocessed using CAR seem to show progressive phase shifts even outside the stimulation period (350 ms before stimulation onset) showing clearly the effects of volume conduction (see Fig. 6). Applying surface Laplacian eliminated this effect and the phase shift was only observed during the stimulation period (see Fig. 4c). Additionally, the source localization analysis, revealing neuronal current generators underlying EEG scalp topographies, confirmed the spread of the observed propagation and was used as a basis for our further analysis.

The sources identified by sLORETA for the fundamental and sub-harmonic components seemed to agree with what has been previously reported^14,56^. We, however, did not find a significant activation for the first harmonic component. A reason for that might be that all our stimulation condition were in the high frequency region, resulting in first harmonic component in the range from 80 to 120 Hz. The decrease in SNR for higher order harmonics may distort the LORETA analysis. Wagner et al. suggest that simultaneously active sources can only be separated well by sLORETA analysis if their fields are distinct enough and of similar strength, and in the context of a strong or superficial source, weak or deep sources remain invisible^57^. In our previous work we did observe a linear decrease in SSVEP strength at the first harmonic especially at higher frequencies^52^. It was encouraging to see that the cortical sources identified by the sLORETA analysis for the first harmonic of our lowest stimulation condition (40 Hz) were consistent with those identified by Pastor et al.^56^ for the same frequency (their highest frequency condition).

Regarding the differences observed for the highest stimulation conditions at 58 and 60 Hz, stimuli at those frequencies at average light level of about 130 cd/m$^2$ and assuming an average pupil diameter of about 4 mm, would fall very close to the visual perception threshold as defined by the temporal sensitivity curve at retinal illuminance of 1000 Td and above^58^. The responses in the high frequency range could possibly have different neural representations and the associated neural mechanisms could be different from those governing conscious visual perception at lower frequencies^11,59^.

Broadband pseudo random noise sequences produce a code-modulated visual evoked potential response (cVEPs) that shares many characteristics with SSVEPs, including topology. This approach has been successful in BCIs^60^. A comparisons with SSVEP would boost insight in the respective mechanisms and the evidence for SSVEP propagation model may well inform research in broadband visual evoked potentials. It is not clear if the processing of periodic versus noise-like stimuli is open to attention modulation in different ways. But such propagation model may point to possible gain in detection methods, also for these stimuli.

While no conceptual framework could possibly account for the complex properties and interactions in the human brain, models considering both the spatial and temporal aspects of the cortical dynamics would provide better representation of the underlining mechanisms.

The broad range of SSVEP applications in research and clinical practice call for reduced complexity, which would positively impact the ease of use, patient throughput, comfort and safety. In this manuscript, we investigated the spatiotemporal oscillatory properties of SSVEPs using a sparse electrode montage and high stimulation frequencies. We show what are the cortical sources underlying HF-SSVEPs as well as their spread and propagation velocity. This work could help building more practical solutions, improve processing speed, reduce erroneous interpretation and enhance the patient experience. Furthermore, this can open new doors for studying the neural dynamics in the gamma band, which functional significance is only starting to be understood.

## Methods

### Participants

For this study we invited 32 healthy volunteers with normal or corrected to normal vision (14 male, 18 female, age range 20–30 years old). Because of the nature of the stimuli, the participants were carefully screened for a history of photosensitivity, seizures, migraine, or headaches and only healthy participants not reporting any of these conditions were enrolled in the study. They were informed about the goals of the study and asked to sign an informed consent before the start of the experiment. The study was approved by the Philips internal ethics committee on biomedical experiments and conducted in accordance with the declaration of Helsinki Guidelines for Ethics in Research.

### Experimental paradigm

**Figure 7:**
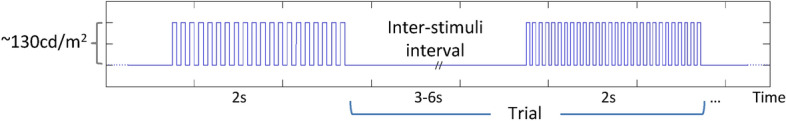
Stimulation pattern.

During the experimental session the participants were instructed to focus their attention on an LED panel with dimensions 44 $\times$ 36 cm, and to avoid eye blinks and movement as much as possible. The panel was placed centrally on a desk at a distance of about 70 cm and spanning a visual angle of about 35$^{\circ }$. The panel consisted of twenty 2 mm white LEDs (Philips Lumileds LXHL) evenly distributed in 4 rows and 5 columns (approx. 8 cm apart in each dimension) covered by a diffusing screen. It delivered repetitive visual stimulation in the form of square waves at 100% modulation depth and 50% duty cycle. The average light output of the stimuli was approx. 130 cd/m$^2$. The experimental conditions comprised RVS at 10 different frequencies from 40 to 60 Hz with a step size of 2 Hz, excluding 50 Hz as it coincides with the power line frequency at the study site. Stimuli presentation was randomized and each condition was presented 10 times, which is expected to improve the SNR by a factor of $\sqrt{(}10) \approx 3$^61^. Each trial consisted of an inter-stimulus interval, where no light was present, of random duration between 3 and 6 s, followed by a 2 s long stimulation period (see Fig. 7). Trials were grouped in blocks of 10 separated by a break of 10 s during which the participants could rest, move or blink. The average experimental duration was approx. 13 min and ensured that effects of fatigue or decreased alertness, which might affect the response^62^, are minimized.

### Data acquisition

BioSemi ActiveTwo signal acquisition system (BioSemi B.V., Amsterdam, The Netherlands) was used to record EEG via 32 sintered Ag/AgCl active electrodes at a sampling frequency of 2048 Hz. The electrodes were mounted on an elastic cap according to the 32-channel extension of the international 10–20 positioning system (see Fig. 4a). The setup used an added common mode sense and driven right leg as “ground” electrodes. The signal from a photo diode was jointly recorded with the EEG to mark the presentation of the stimuli.

### Data analysis

#### EEG preprocessing

The recorded signals were analysed using custom MATLAB scripts and the open source EEGLAB toolbox^63^. First, a 50 Hz notch filter was applied to attenuate the power line interference. Eye-blinks and other ocular artifacts were removed using independent component analysis (ICA)^53^ by excluding the component showing the highest average spatial difference between front and back electrodes. The signals were subsampled to 256 Hz to reduce the computation time, and surface Laplacian^64,65^ was applied to reduce the effect of widely distributed background neuronal activity and to enhance the spatial localization of the signals.

The signals were then segmented into non-overlapping epochs time-locked to stimulus onset. The epochs started 1000 ms before stimulus onset and ended 3000 ms after, including 2000 ms of stimulation and a 1000 ms long period after stimulus offset.

#### Time frequency decomposition

All preprocessed epochs were subjected to a time-frequency decomposition using the fast Fourier transform (FFT) with a 126 samples (500 ms) wide Hanning window and 4.5 samples (17.59 ms) overlap (a total of 200 windows). The average power at each stimulation frequency was, then, calculated for five 250 ms-wide windows, starting with a baseline interval 250 ms before and continuing for 1000 ms after stimulation onset. Using this estimate we define the event related synchronization (ERS) as the fraction power change in each 250 ms window referenced to the 250 ms baseline interval. The resulting dynamics in each channel were then visualized on a topographic map. For this analysis the window length and baseline were optimized to improve the visual representation of the spatial maps.

#### Source localization

We estimated EEG cortical source current density with low-resolution brain electromagnetic tomography using the sLORETA software^66^. sLORETA calculates the standardized current source density (CSD) in the gray matter and the hippocampus at each of the 6239 voxels of the Montreal Neurological Institute (MNI) reference brain. CSD at the frequency of stimulation and its harmonics for each condition was estimated using a regularization factor of 10 (SNR = 10). For this analysis we computed the average cross spectra for each condition over 1 second long epochs starting 250 ms after stimulus onset. The results were then compared to the 1 second period before stimulation onset using sLORETA’s built-in non-parametric statistical analyses (statistical non-parametric maps, SnPM) using log-F-ratio statistic for paired groups with a threshold of $p < 0.01$ and $p < 0.05$ and permutation test repeated 5000 times, correcting for multiple comparisons.

#### Phase synchrony analysis

Phase synchrony between two signals was estimated by looking for latencies at which the phase difference between signals varies little across trials^67^. For this analysis, all signals including the signal from the photo diode monitoring the light stimulation, were first filtered with a band-pass Parks–McClellan finite impulse response (FIR) filters centered around each stimulation frequency ($Fstop: F \pm 3 Hz, Fpass: F \pm 1 Hz, Dpass: 0.058, Dstop: 10^{-4}, density factor = 20$). The filter properties were chosen such that the filter is stable, while ensuring a narrow bandpass response. FIR filters were chosen because they can be designed to have an exact linear phase response. The constant group delay after filtering was estimated and signals were corrected for it.

Next, the instantaneous phase of the signal *y*(*t*) was computed using the Hilbert transform^68^. The Hilbert transform $y_h(t)$ is the convolution of the signal *y*(*t*) with the function $h(t)=1/\pi t$ such that:

1 $$\begin{aligned} y_h(t)=\frac{1}{\pi } \int _{-\infty }^{\infty } y(\tau )h(t-\tau ) d\tau , \end{aligned}$$

where $\tau$ is the integration variable.

The Hilbert transform enables the analytical representation of the signal as follows:

2 $$\begin{aligned} Y(f,t)=y(t)+j \cdot y_h(t) = A_y(t) e^{j\phi _y(t)} \end{aligned}$$

Comparison of the Hilbert transform and the FFT-based method revealed no substantial difference in phase estimates.

The phase difference $\Delta \phi _{12}(t)$ between two time series $y_1(t)$ and $y_2(t)$ with instantaneous phases $\phi _1(t)$ and $\phi _2(t)$, respectively, was calculated as follows:

3 $$\begin{aligned} \Delta \phi _{12}(t)=arg(e^{j(\phi _1(t)-\phi _1(t))}) \end{aligned}$$

The phase locking value (PLV) for each condition, channel and subject at time *t* was then defined as the average value over trials ($n = 1,\ldots ,N$):

4 $$\begin{aligned} PLV(t)=\frac{1}{N} \left| \displaystyle \sum _{i=1}^{N} e^{j\Delta \phi (t, n)}\right| \end{aligned}$$

The phase locking statistics (PLS)^67^ measures the significance of the phase covariance between the two signals. It is a statistical test based on a surrogate data aiming at differentiating significant PLVs against noise. PLS was calculated by shuffling the trials for another randomly selected stimulation condition and calculating the respective surrogate PLV. The PLS comprises the proportion of the surrogate values higher than the original PLV. We chose $PLS \le 5\%$ to ensure the probability of having false positive rate below $5\%$.

#### Distance estimates

The distance $\Delta \delta _{12}$ between electrode locations was estimated as the great-circle distance between two points on a sphere using the haversine formula (see Eq. (5)). We assumed a spherical head model with a radius of 9 cm.

5 $$\begin{aligned} \begin{aligned} haversin(\alpha )&= \sin ^2\bigg (\frac{\phi _1 - \phi _2}{2}\bigg ) + \cos (\phi _1)\cos (\phi _2)\sin ^2\bigg (\frac{\lambda _1 - \lambda _2}{2}\bigg ) \\ \Delta \delta _{12}&= 2 r \arcsin (\sqrt{haversin(\alpha )}), \end{aligned} \end{aligned}$$

where *r* is the radius of the sphere; $\phi _1, \phi _2$ are the latitudes of the two points in radians; and $\lambda _1, \lambda _2$ are the longitudes of the two points in radians.

#### Phase velocity

Phase velocity *v* is the speed at which the phase of a wave propagates over the scalp and is calculated as follows:

6 $$\begin{aligned} v(t) = 2 \pi f (\Delta \delta _{12}/\Delta \phi _{12}(t)), \end{aligned}$$

where *f* is the stimulus frequency, and $\Delta \phi _{12}(t)$ is the phase difference as defined in Eq. (3) and $\Delta \delta _{12}$ is distance between electrodes as defined in Eq. (5).

## Data Availability

The data that support the findings of this manuscript are not publicly available, as it will potentially compromise research participant consent under the European GDRP law regarding reuse of data for secondary research and development purposes.

## Supplementary Informtaion

Supplementary Informtaion 1.
