# Supplementary material for: SSVEP phase synchronies and propagation during repetitive visual stimulation at high frequencies
Source: Sci Rep. 2021 Mar 2;11:4975. doi: 10.1038/s41598-021-83795-9 (PMC7925656; doi:10.1038/s41598-021-83795-9)
Supplement: Supplementary file 1 — Supplementary Informtaion 1. [file 41598_2021_83795_MOESM1_ESM.pdf]

Supplementary Infomation

SSVEP phase synchronies and propagation during repetitive visual stimulation at high frequencies

Tsvetomira Tsoneva[1,2,*], **Gary Garcia-Molina**[3,4], **and Peter Desain**[2]

[1]Brain, Behaviour and Cognition department, Philips Research, High Tech Campus 34, 5656 AE, Eindhoven, The Netherlands
[2]Donders Institute for Brain, Cognition and Behaviour: Centre for Cognition, Radboud University, Montessorilaan 3, 6525 HR Nijmegen, The Netherlands
[3]Sleep Number Labs, 111 N Market St, San Jose, 95113 California, USA
[4]Center for Sleep and Consciousness, University of Wisconsin, Madison, 53719 Wisconsin, USA
[*]tsvetomira.tsoneva@philips.com

Table S1. Log-F-ratio thresholds for the sub-harmonic component at each stimulation condition

Stimulation condition	F($p < 0.01$)	F($p < 0.05$)
40	0.798	0.692
42	1.018	0.951
44	0.978	0.900
46	0.735	0.678
48	1.044	0.968
52	1.110	1.030
54	1.512	1.446
56	1.299	1.267
58	1.069	1.018
60	1.183	1.039

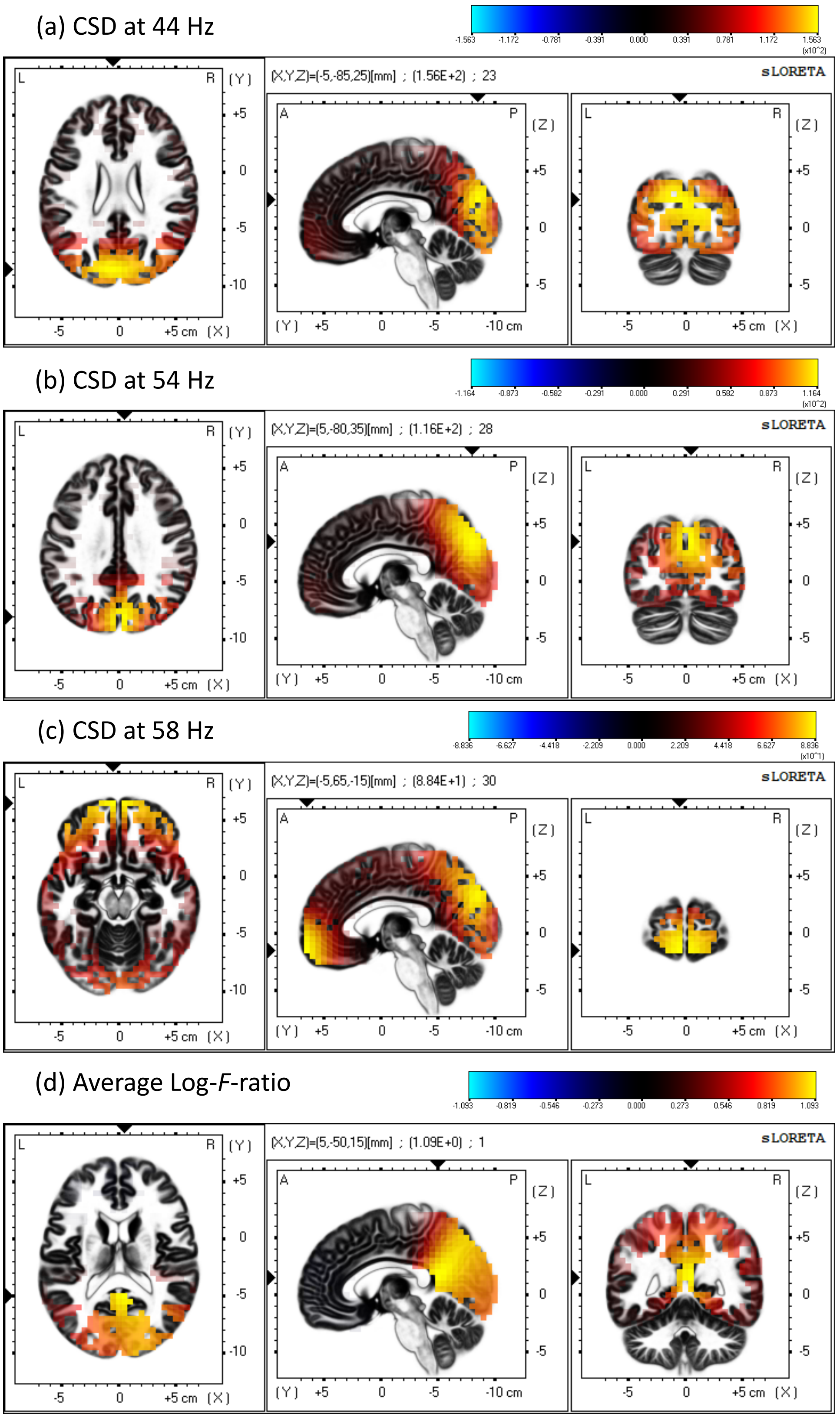

Figure S1. Low-resolution brain electromagnetic tomography (sLORETA) at the sub-harmonic frequency component (1/2f) under diverse stimulation conditions: (a) 44 Hz, (b) 54 Hz, (c) 58 Hz. (d) The average log F-ratio statistics across all sub-harmonic frequency components. The squared magnitude of the CSD and the average log F-ratio are colour coded from blue (minimum) through grey (zero) to red and bright yellow (maximum). Slices from left to right: axial (viewed from top), sagittal (viewed from left), and coronal (viewed from back). L, left; R, right; A, anterior; P, posterior.
